# The Tuning of Optical Properties of Nanoscale MOFs-Based Thin Film through Post-Modification

**DOI:** 10.3390/nano7090242

**Published:** 2017-08-29

**Authors:** Wenchang Yin, Cheng-an Tao, Xiaorong Zou, Fang Wang, Tianlian Qu, Jianfang Wang

**Affiliations:** 1 College of Science, National University of Defense Technology, Changsha 410073, China; yinwenchang8@163.com (W.Y.); Xr5229@163.com (X.Z.); wangaaf02@163.com (F.W.); 2 College of Optoelectronic Science and Engneering, National University of Defense Technology, Changsha 410073, China; qutianliang@nudt.edu.cn

**Keywords:** metal-organic frameworks, thin film, optical property, refractive index, post-modification

## Abstract

Optical properties, which determine the application of optical devices in different fields, are the most significant properties of optical thin films. In recent years, Metal-organic framework (MOF)-based optical thin films have attracted increasing attention because of their novel optical properties and important potential applications in optical and photoelectric devices, especially optical thin films with tunable optical properties. This study reports the first example of tuning the optical properties of a MOF-based optical thin film via post-modification. The MOF-based optical thin film was composed of NH_2_-MIL-53(Al) nanorods (NRs) (MIL: Materials from Institute Lavoisier), and was constructed via a spin-coating method. Three aldehydes with different lengths of carbon chains were chosen to modify the MOF optical thin film to tune their optical properties. After post-modification, the structural color of the NH_2_-MIL-53(Al) thin film showed an obvious change from purple to bluish violet and cyan. The reflection spectrum and the reflectivity also altered in different degrees. The effective refractive index (*n*_eff_) of MOFs thin film can also be tuned from 1.292 to 1.424 at a wavelength of 750 nm. The success of tuning of the optical properties of MOFs thin films through post-modification will make MOFs optical thin films meet different needs of optical properties in various optical and optoelectronic devices.

## 1. Introduction

Metal organic frameworks (MOFs) are crystalline porous organic-inorganic hybrid materials that consist of metal ions or clusters coordinated to organic ligands to form one-, two-, or three-dimensional structures [1,2]. They have attracted immense attention due to their outstanding features, such as ultrahigh porosity [3], large internal surface area [4], good thermal stability [5] and high chemical tailorability [6]. These properties make MOFs promising candidates for applications in gas storage [7,8], catalysis [9], drug delivery [10,11], semiconductors [12,13] and chemical sensing [14,15,16,17,18,19,20,21]. In order to impart sophisticated and nuanced chemical and physical properties to MOF materials, different functional groups should be introduced into MOFs. However, the preparation of highly functionalized MOFs has been largely limited to direct synthetic methods. Fortunately, post-synthetic methods have become an efficient way to achieve the chemical modification of MOFs due to the abundant and ready reactions of linkage ligand molecules [6].

Optical properties, which determine the application of optical devices in different fields, are the most significant properties of optical thin films. Recently, MOF-based thin films, in particular, optical thin films, have attract more and more attention, since they can potentially be used as active components (which can be easily regulated) in functional devices [22,23,24,25,26,27]. This is in contrast to the difficulty of tailoring the optical properties of conventional dense inorganic optical films. In 2010, G. Férey and coworkers [28] first described a simple route for the preparation of MIL-89 optical thin films by dip-coating. Then, they reported MIL-101(Cr) [29], MIL-100(Cr) [30] and MIL-100(Fe) [30] optical thin films. Redel et al. [31] presented the optical properties of surface-anchored metal-organic frameworks. Lu et al. [17] constructed zeolitic imidazolate framework (ZIF)-8-based Fabry-Pérot devices that function as selective sensors for chemical vapors and gases. Wu and coworkers [16] also fabricated three-dimensional MOF-based photonic thin films for vapor sensing. Hinterholzinger et al. [14] reported the first MOF-based one-dimensional photonic crystal (1DPCs), in which ZIF-8 was used as the active component. Ranft et al. [32] constructed tandem MOF-based photonic crystals for enhanced analyte-specific optical detection. Liu et al. [33] fabricated monolithic surface-anchored MOF Bragg reflectors for optical sensing. To improve the spectral shift of the photonic film when exposed to analyte vapors, in 2014, our group [18,34] first fabricated flexible MOF-based photonic films for sensing. Recently, Müller et al. [35] fabricated a photoswitchable nanoporous film by loading azobenzene in metal–organic frameworks of a type whose optical properties can be tuned by absorbed photo-responsive guests. The optical properties of MOF thin films can be changed by adsorbing different chemical vapors; however, it is impossible to sustain for an adequately long time for real uses in photonic devices. It is known that post-synthetic methods can cause a permanent and stable change of the ligands of MOFs, but to our knowledge, there is little research on tuning the optical properties of MOF-based optical thin films via such a facile method.

**Scheme 1:**
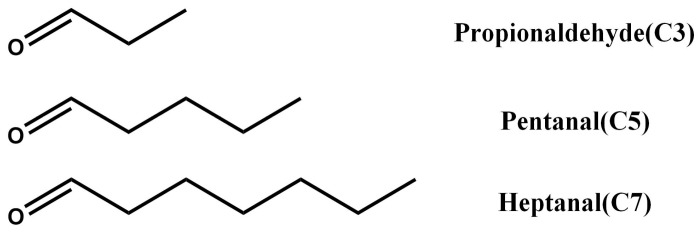
Molecular structures of aldehydes.

In this study, a post-modification method for tuning the optical properties of MOF thin films has been developed. A well-known MOF, NH_2_-MIL-53(Al) (MIL: Materials from Institute Lavoisier), composed of Al(III)-O clusters connected by 2-aminoterephthalic acid (H_2_N-BDC) units, were chosen as the model, and nanoscale NH_2_-MIL-53(Al) nanorods (NRs) were hydrothermally synthesized. The MOF optical thin film was fabricated by spin-coating alcoholic suspensions of NH_2_-MIL-53(Al) NRs onto the substrate. Three aldehydes with different lengths of carbon chains (Scheme 1) were chosen to tune the optical properties of the MOF thin films through the reaction with amine groups of MOFs. After post-modification, the color of the thin film showed an obvious change from purple to bluish violet and cyan. The reflection spectrum and the reflectivity also altered to different degrees. The effective refractive index (*n*_eff_) of the MOF thin film was readily tuned from 1.292 to 1.424 at the wavelength of 750 nm. The success of post-modification and the degree of modification were characterized by X-ray photoelectron spectroscopy (XPS). The success of tuning of the optical properties of MOF thin film through post-modification will make MOF optical thin films meet the different required optical properties in various optical and optoelectronic devices.

## 2. Experimental and Computational Method

### 2.1. Materials

2-Aminoterephthalic acid (H_2_N-BDC, 99%), Al(NO_3_)_3_·9H_2_O (97%) was purchased from J&K chemical (Beijing, China); propionaldehyde (C3), pentanal (C5) and heptanal (C7) were obtained from Macklin Biochemical Co., Ltd. (Shanghai, China). Other chemicals were provided by a local supplier. All chemicals and solvents were used as received.

### 2.2. Preparation of NH_2_-MIL-53(Al) Optical Thin Film

NH_2_-MIL-53(Al) nanorods (NRs) were hydrothermally synthesized by adding sodium acetate according to the literature [36], with modification. The suspensions of NH_2_-MIL-53(Al) NRs were obtained by dispersing the NH_2_-MIL-53(Al) NR powder in ethanol (99.9%). The substrates were silicon wafers (2 cm × 2 cm) cleaned by detergent solution and piranha solution (H_2_SO_4_/H_2_O_2_, volumetric ratio 7:3). The wafers were dried in an oven after thoroughly rinsing with deionized (DI) water. After that, the optical thin films were fabricated by spin-coating 200 μL of NH_2_-MIL-53(Al) NRs in alcoholic suspensions (5.0 wt %) onto the substrate. The rotation speed was maintained at 2800 rpm for 60 s. Then, the films were heated in an oven at 200 °C for 20 min. 

### 2.3. Post-Modification of NH_2_-MIL-53(Al) Optical Thin Film

One piece of the optical thin film was cut into four equal slices using a diamond cutter. Then, the slices were put into a tubular furnace at 330 °C for 6 h before post-modification [27]. Three of the equal slices were immersed in a toluene solution of C3, C5, or C7 (0.1 M) at 90 °C for 12 h for post-modification. After completion of the reaction, the optical thin films were rinsed with toluene three times and dried in a vacuum at room temperature. For comparative analysis, the last remaining piece of the MOF film was soaked in pure toluene at the same time.

### 2.4. Characterization

Fourier transform infrared (FT-IR) spectra for the bulk material were determined using a Spectra Two spectrophotometer (PerkinElmer, Waltham, MA, USA) from 4000 cm^−1^ to 400 cm^−1^ with attenuated total reflection (ATR) accessory. Transmission electron microscopy (TEM) micrographs were obtained using a JEOL JEM 1230 electron microscope (JEOL Ltd., Tokyo, Japan) with an accelerating voltage of 200 kV. Size distribution was measured using a NICOMP 380 partical size analyzer (Particle Sizing Systems, Orlando, FL, USA). X-Ray Diffraction (XRD) patterns of powders were collected using a Ttr III type X-ray diffractometer (Rigaku, Tokyo, Japan). Data were collected between 5° and 50° with a graphite-monochromated CuKα radiation source. Thermogravimetric analysis (TGA) was completed by a STA6000 thermogravimetric analyzer (Perkin Elmer, Waltham, MA, USA) at a scanning rate of 5 °C min^−1^ under N_2_ atmosphere from 30 to 700 °C. The N_2_ adsorption-desorption isotherm of the samples at liquid nitrogen temperature (78 K) and gas saturation vapor tension range were measured by a BEL Mini sorption instrument (MicrotracBEL, Tokyo, Japan). Contact angles were measured using a JC-2000X1 (Shanghai zhongchen digital technic apparatus co ltd., Shanghai, China) contact angle analyzer. Scanning electron microscopy (SEM) images were obtained by a Quanta FEG250 electron microscope (FEI Ltd., Hillsboro, OR, USA). The samples were sputtered with a thin layer of Au prior to imaging. The XPS measurements were performed with a K-Alpha 1063 (Thermo Fisher Scientific, Waltham, MA, USA); XPSPEAK v4.0.0.0 software (CUHK, Hongkong, China) was used to perform curve fitting. Ellipsometry measurements used a XLS-100 ellipsometer (J.A.Woollam, Lincoln, NE, USA) at an angle of 65°, 70° and 75° and within a spectral range of 400–1000 nm at room temperature. ^1^H Nuclear Magnetic Resonance (NMR) experiments were performed on Agilent DD2 400 (Agilent, Santa Clara, CA, USA).

## 3. Results and Discussion

### 3.1. Preparation and Characterization NH_2_-MIL-53(Al) NRs

**Figure 1:**
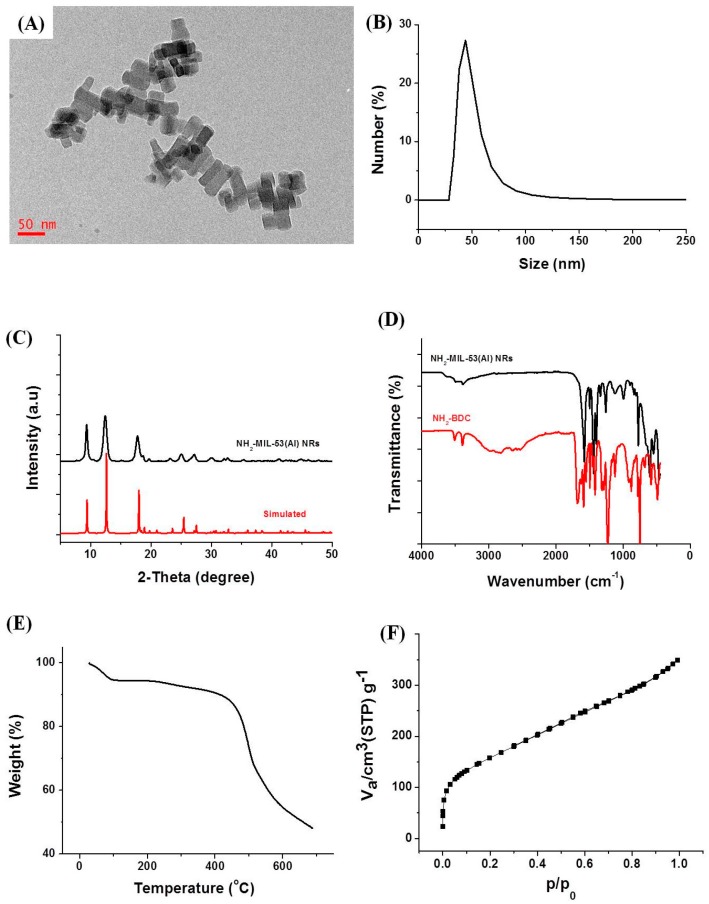
TEM (**A**), DLS (**B**), XRD (**C**), FT-IR (**D**), TGA (**E**) and N_2_ ads-des isotherms (**F**) of NH_2_-MIL-53(Al) NRs.

As shown in Figure 1A, NH_2_-MIL-53(Al) NRs of high quality in terms of their size were obtained according to the method reported by J. M. Chin et al. [36]. The particle sizes in length and width were about 44.8 ± 13.2 nm and 23.4 ± 3.8 nm, respectively, based on the TEM (Figure 1A and Figure S1). The particle size in ethanol was 43.8 nm, as measured by dynamic light scattering (DLS) method (Figure 1B). The length of the MOF NRs was shorter than that of previous report (107.5 ± 73.3 nm) [36], because of the reduction of sodium acetate from 5 times of Al(NO_3_)_3_·9H_2_O to 3 times. The NRs were further characterized by XRD, FT-IR, TGA and N_2_ adsorption-desorption isotherms (Figure 1C–F). The observed powder XRD pattern of NH_2_-MIL-53(Al) NRs was in good agreement with the simulated pattern [37], which confirms the formation of pure NH_2_-MIL-53(Al). Compared with the FT-IR spectrum of NH_2_-BDC, the bands of the protonated carboxylic groups at around 1710 cm^−1^ disappeared, indicating all of the carboxyl groups were coordinated with Al^3+^. NH_2_-MIL-53(Al) NRs exhibited good thermal stability up to 420 °C, which was consistent with a previous report [38]. The BET area of the NRs calculated between P/P_0_ 0.01 and 0.25 was 560 m^2^/g. Accordingly, the total pore volume of NRs was 0.54 cm^3^/g. These results indicated that nanoscale NH_2_-MIL-53(Al) had been successfully prepared, and that NH_2_-MIL-53(Al) NRs have abundant pores and large specific surface area. This benefits the following modification.

### 3.2. Preparation of NH_2_-MIL-53(Al) Optical Thin Film

**Figure 2:**
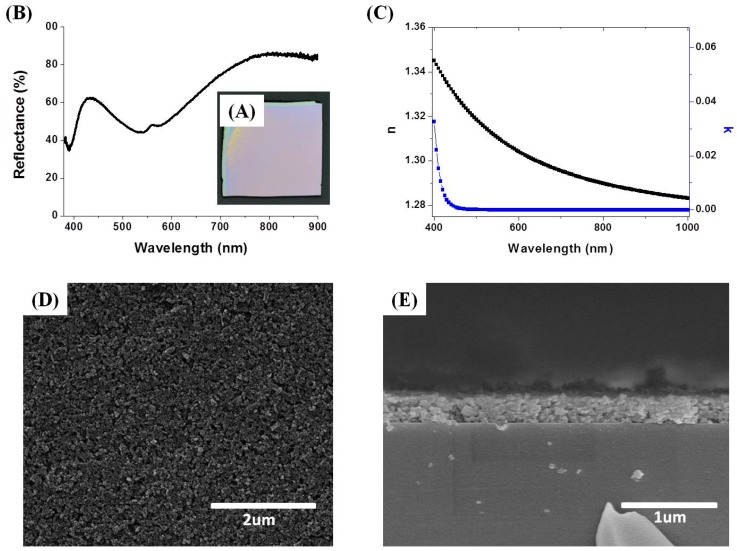
Photograph (**A**), Reflection spectrum (**B**), RI (*n*) and extinction coefficient (*k*) (**C**), SEM image ((**D**) top-view, (**E**) side-view) of MOF optical thin film.

The MOF-based optical thin films were fabricated by a spin-coating method according to our previous reports [18,34]. A photograph of an as-prepared MOF NR optical film is shown in Figure 2A. It shows a vivid purple color. The specular reflection spectrum of NH_2_-MIL-53(Al) optical thin film was evaluated as shown in Figure 2B. There is a Bragg peak centered at 430 nm, and the reflectivity is about 70%. The effective refractive index (*n*_eff_) and extinction coefficient (*k*) of MOFs thin film was evaluated with ellipsometry from 400 nm to 1000 nm, and the obtained values for the MOF thin film as a function of wavelength are shown in Figure 2C. The prepared MOF thin film has an optical constant of *n* = 1.291 and *k* ≈ 0 at 750 nm. The SEM images (Figure 2D,E) show the NR optical thin film with a smooth surface and a uniform thickness of 301.3 ± 11.7 nm. It can be seen that there are some tiny gaps in the surface, which may be induced by tensile stress during the thermal annealing process. The low refractive index (RI) were mainly induced by the high intrinsic porosity of NH_2_-MIL-53(Al), and were also affected by the inter-voids between the NRs. 

### 3.3. Tuning of Optical Properties of NH_2_-MIL-53(Al) Optical Thin Film

**Figure 3:**
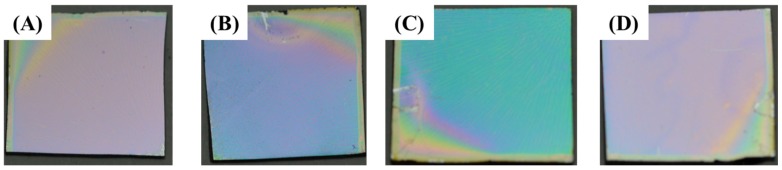
Photographs of MOF optical thin film without modification (**A**), modified with propionaldehyde (**B**), pentanal (**C**) and heptanal (**D**).

It is known that the amino group of the ligand NH_2_-BDC has a large number of chemical reactions, as the aldehyde can react with amino to form imide easily. Aldehyde molecules with varied carbon chain lengths—propionaldehyde (C3), pentanal (C5) and heptanal (C7)—were selected to modify the optical properties of NH_2_-MIL-53(Al) optical thin film. Photographs of MOF optical thin film without modification and after modification are shown in Figure 3A–D. The vivid structural color of the thin film changed from purple to bluish violet and cyan after modification with C3 and C5, but only changed a little after C7 modification, suggesting that C7 molecules do not efficiently react with the aminos of MOFs.

**Figure 4:**
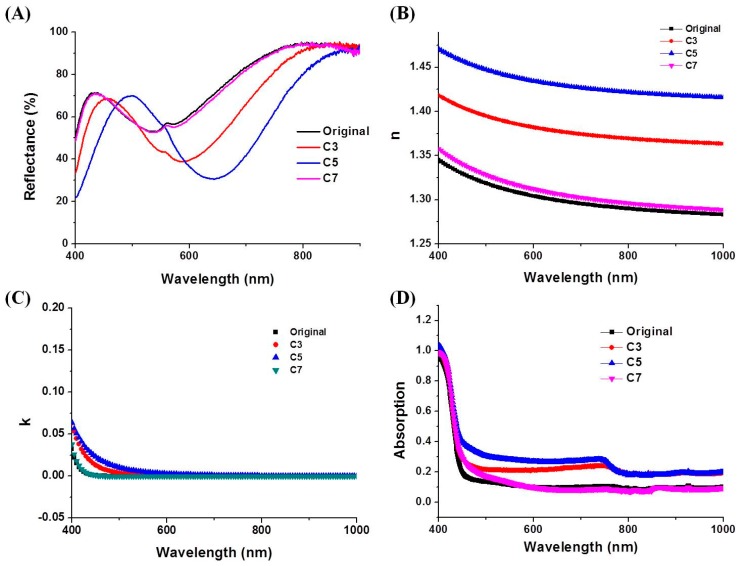
Reflection spectra (**A**), the effective refractive index (*n*) (**B**), the extinction coefficient (*k*) (**C**) of MOF optical thin films and diffuse reflectance spectra of MOF powders (**D**). C3, C5, C7 represent the MOF optical films modified with propionaldehyde (C3), pentanal (C5), and heptanal (C7), respectively.

The specular reflectance spectrum of the optical film can be tuned after post-modification. As shown in Figure 4A, the reflectance spectrum shifted to the red edge for all cases. The shift of the Bragg peaks after C5 modification was larger than that after C3 modification, and was also larger than that after C7 modification. The Bragg peaks red shifted from 433 nm to 498 nm after C5 modification, and accordingly, the reflectance at 641 nm decreased from 70% to 30%. The trends in the spectral shift are consistent with that of the color change. 

In actual optical or opto-electronic devices, the requirement for effective refractive indices of optical thin films is specific. Therefore, using the diversity of organic synthetic chemistry, the RI of the optical films regulated by post-modification with rational molecules is significant. In the present work, aldehyde molecules with varied carbon chain lengths—propionaldehyde (C3), pentanal (C5) and heptanal (C7)—were chosen as the modifier. The tuning effect of the modification on the effective RI of the optical thin films was explored by ellipsometry (Figures S2–S5). The obtained *n*_eff_ for the MOF thin film as a function of wavelength are shown in Figure 4B. For convenience of discussion, we chose the index values at the wavelength of 750 nm for all samples. After modification with C3 and C5, the *n*_eff_ of the optical thin films increased proportionally with the length of the carbon chain, from 1.292 (original) to 1.371 (C3) and 1.424 (C5). The RIs of liquid C3 and C5 were only about 1.36 and 1.39, respectively. Such large increases of *n*_eff_ MOF thin films were not only due to the increase in the alkyl chain in the inner pore of NH_2_-MIL-53(Al), but can also be attributed to the change in the electronic structure induced by modifiers [31]. After the modification with C7, only a minor change of *n*_eff_ occurred (From 1.292 to 1.299). The change trend of *n*_eff_ was consistent with the results of the color and reflection change. The molecular size of C3, C5, and C7 were estimated to be 5.5 Å, 8.1 Å, and 10.7 Å, respectively. It is known that NH_2_-MIL-53(Al) frameworks possess a diamond-shaped one-dimensional channel, whose free diameter is close to 8.5 Å (Figure S6). The amino groups locate at the wall of the channel and are parallel to the channel (Figure S7). When the aldehyde molecules have reacted with the amino, the alkyl chain would stretch into the channel, as illustrated in Figure S8. The size of the channel is big enough for the C3 and C5 molecules, but the chain of C7 must curl to fit into the channel, which will limit the reaction of C7 with amino. From the crystal structure of NH_2_-MIL-53(Al), there are two amino groups around one channel in a unit cell, but the volume of the channel can only hold one C3 or C5 molecule, so the maximum degree of modification that can be achieved is 50%. In the case of C7 modification, only one quarter of the amino can be modified, due to the steric hindrance. This is the reason that only a small change of the optical properties of MOF thin film was observed after the modification with C7.

Additionally, the extinction coefficient (*k*), the imaginary part of the complex refractive index, is another important parameter for optical films. A higher value of *k* indicates a greater amount of attenuation when the light propagates through the material. When *k* equals 0, the material becomes transparent in the according range. The evaluated *k* values of the MOF thin films are shown in Figure 4C. The *k* of the original MOF film decreases from 400 nm to 450 nm, and decreases to 0 when the wavelength is longer than 450 nm, up to 1000 nm. It means that MOF thin films can serve as a transparent material in the range from 450 to 1000 nm. After effective post-modification, the extinction coefficient becomes remarkable. On the one hand, the value of *k* at a wavelength of 450 nm increases from 0.001 to 0.013 (C3) and 0.023 (C5); on the other hand, the cut-off wavelength (*k* < 0.001) shifted from 450 nm to 570 nm (C3) and 735 nm (C5). After modification with C7, nearly no change of *k* occurred. These results suggest the the band structure of the MOFs have changed following modification. To prove this, the diffuse reflectance spectra of MOF powders before and after modification were taken and transformed to absorption spectra, as shown in Figure 4D. Obviously, the absorption of the MOFs was enhanced, whatever the increased value or the extended cut-off wavelength, which agreed with the results of *k*. The bandgap of the MOFs was calculated from the plots of (Ahν)^1/2^ versus hν (Figures S9–S12). It decreased from 2.74 eV to 2.71 eV (C3) and 2.68 eV (C5). The trend is coincident with that of *k*. 

**Figure 5:**
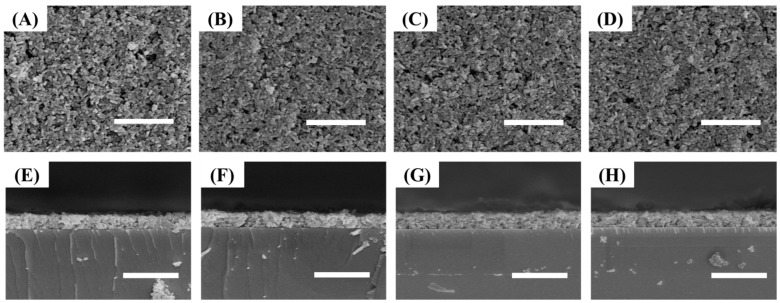
SEM images ((**A**–**D**) top-view, (**E**–**H**) side-view) of MOF optical films without modification (**A**,**E**), modified with propionaldehyde (**B**,**F**), pentanal (**C**,**G**) and heptanal (**D**,**H**). (1 bar = 1 μm).

The MOF thin films are of great stability. The quality of the thin film after modification was confirmed by SEM observation. As shown in Figure 5A–D, the NRs were all in a disorderly distribution, and there was no obvious change in the surface, which indicates that the optical thin film is very stable after modification. From a side view, the thicknesses of the modified films exhibited very little change (Figure 5E–H). The measured thicknesses of the original film, the modified film with C3, C5, C7 were 292.3 ± 15.7 nm, 296.5 ± 13.3 nm, 312.9 ± 12.4 nm and 303.4 ± 9.7 nm, respectively. The corresponding thicknesses evaluated via ellipsometry were 310.7 ± 0.8 nm, 328.5 ± 0.3 nm, 336.9 ± 1.0 nm and 316.9 ± 2.0 nm, respectively. The variation tendency of the thickness agrees with each other. The roughness of the thin films led the ellipsometry method to overestimate their thickness. The slight change of the thicknesses is on account of the change in crystal structure of NH_2_-MIL-53(Al) caused by the post-modification.

To confirm the success of the modification, the FT-IR of MOFs without modification, modified with propionaldehyde (C3), pentanal (C5) and heptanal (C7) were taken, as shown in Figure S13. The peaks around 3500 cm^−1^ and 3400 cm^−1^ are attributed to the stretching vibration of N–H. It is hard to make out the decrease of the NH stretches due to the low degree of reaction of the amino. The shoulder peak around 1600 cm^−1^ is ascribed to the N=C stretch, but it is not significant, because of the overlap of the bands of the C=C stretch. The bands of the alkyl groups from 2800 cm^−1^ to 3000 cm^−1^ (the dashed line in Figure S13B) appear after post-modification, indicating that there are alkyl groups in post-modified MOFs. In addition, the lack of peaks around 1730 cm^−1^, which is attributed to the C=O stretches (Figure S14), suggests that there are no free aldehyde molecules confined in the channels of the MOFs.

**Figure 6:**
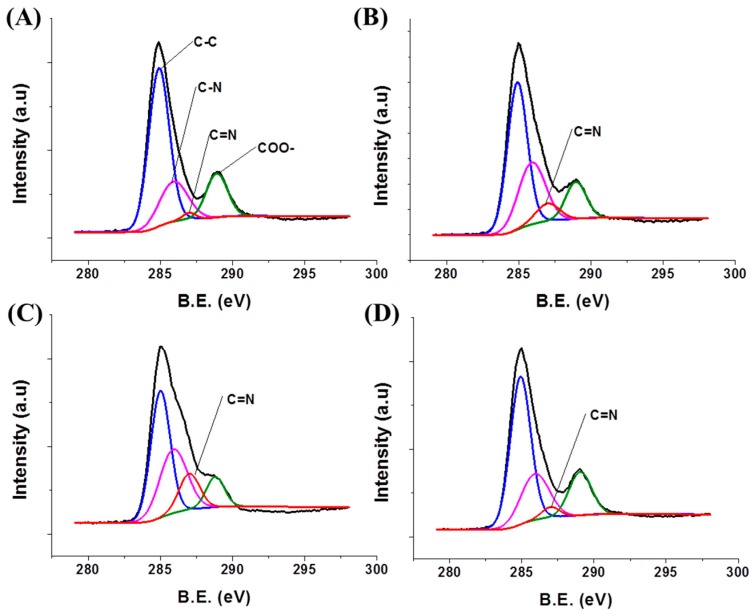
The C1s XPS spectra for MOF optical films without post-modification (**A**), modified with C3 (**B**), C5 (**C**) and C7 (**D**).

Furthermore, the degree of modification of the optical thin film was evaluated by the XPS measurement. The C1s XPS spectra of modified NH_2_-MIL-53(Al) are optimized by a Gaussian-Lorentzian peak shape. As the XPS is not convincing, the C1s spectra were firstly fitted with only three possible peaks: the non-oxygenated ring C (C–C, 284.9 eV), the C in C–N bonds (C–N, 285.9 eV), and the carboxylate C (COO^−^, 288.9 eV), as shown in Figure S15. The relative atomic percentage of C in the three chemical groups is listed in Table S1. After the post-modification, the ratio of the C in C–N to that in COO^−^ increased significantly. The amount of C in COO^−^ is constant, the sharp increase of the C in C–N (up to 6 times than that in COO^−^) is unreasonable. Assuming the success of modification, the C atoms in modified NH_2_-MIL-53(Al) have four states, according to its different functional groups: the non-oxygenated ring C (C–C, 284.9 eV), the C in C–N bonds (C–N, 285.9 eV), the C in C=N double bonds (C=N, 287.0 eV), and the carboxylate C (COO^−^, 288.9 eV). After the post-modification, the relative atomic percentage of C in C=N increased. Theoretically, if all of the amino groups of ligands (NH_2_-BDC) have reacted with aldehydes to form imines (–C=N–BDC), the relative atomic percentage of C in C=N will equal those in C–N. Thus, the grafting rates of the modification can be estimated by the ratio of C=N to C–N. As the fitted C1s XPS spectra shown in Figure 6 demonstrate, the grafting rates of C3, C5, and C7 modification were about 25.3%, 42% and 14.6%, respectively. The changes of the grafting rates are consistent with the changes of the optical properties, as well as the contact angle, which confirmed the success of the post-modification. This proved the mechanism of post-modification; the aldehyde group reacted with the amino of the ligand NH_2_-BDC to form an imine C=N. In addition, it is expected that a precise control of the optical properties of thin films can be realized by controlling the modification reaction time to reduce the grafting rates.

**Figure 7:**
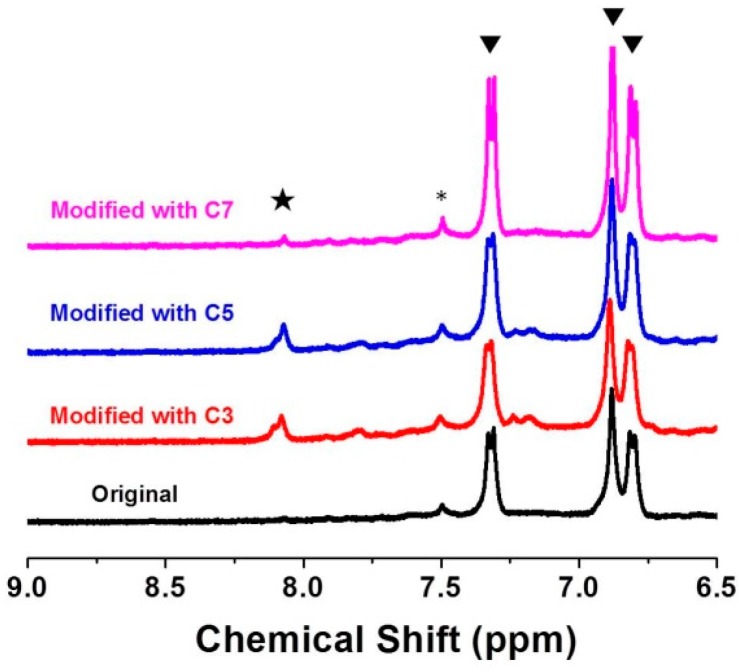
^1^H-NMR spectra of MOFs digested in NaOD-D_2_O solution. From bottom to top: Original MOFs, modified with C3, C5 and C7. Solid pentastars (★ ) and triangle (▼ ) represent signals of hydrogen bonded to imine (–N=C–H) and benzene, respectively.

Finally, ^1^H NMR digestions experiments were performed to quantify the degree of modification. Samples of NH_2_-MIL-53(Al) were digested using sodium deuteroxide (NaOD) solution (3% in deuteroxide, D_2_O), and analyzed by ^1^H-NMR spectroscopy, which confirmed the presence of the modified ligand via the new peak at about 8.07 (Figure 7), which is attributed to the hydrogen bonded to imine (–N=C–H). The absence of the peak at about 9.75 (corresponding to the hydrogen in –CHO group, Figure S16) of modified MOFs suggests again there are no free aldehydes absorbed in the MOFs. The aromatic resonances of the benzene dicarboxylate starting material (NH_2_-BDC) (Figure 7) and the alkyl chains (Figure S17) were used to calculate the yield for each post-modification reaction. The results are summarized in Table S2. The conversion of the amine groups in NH_2_-MIL-53(Al) to imine groups by each aldehyde varied significantly with the length of substituent. Short-chain alkyl aldehydes (C3 and C5) showed high conversion yields of 47.2% and 50.9% for C3 and C5, respectively. In contrast, C7 showed only a 28.0% conversion. These results are very consistent with the aforementioned maximum evaluated from the crystal structure of the MOFs. The degree of modification determined with ^1^H NMR is much more than that determined with XPS, but the trend of the degree of modification is in accordance. 

## 4. Conclusions

The MOF-based optical thin films with a uniform color were fabricated by spin-coating using a NH_2_-MIL-53(Al) NR solution. The color of the optical thin films can change from purple to cyan, and the corresponding reflection peak can red shift from 433 nm to 498 nm, and the reflectance can also decrease to a different degree after the modification. The key optical parameter refractive index can be tuned between 1.292 to 1.424 via modification with different aldehyde molecules. This is because the aldehyde molecules diffused into the inner pore of NH_2_-MIL-53(Al) to react with the amino of MOF ligands. In order to realize a precise regulation of the optical properties, the grafting rate can be regulated by controlling the modification reaction time. According to this study, MOF-based optical thin films with tunable optical properties will meet the different requirements of optical devices and have great potential applications in optical fields. 

## Supplementary Materials

The following are available online at http://www.mdpi.com/2079-4991/7/9/242/s1, Experimental details, statistical histograms of sizes of MOF nanorods, generated and experimental Psi & Delta plots for MOF optical films before and after being modified, FT-IR and ^1^H-NMR spectra.
