# Supplementary material for: The Tuning of Optical Properties of Nanoscale MOFs-Based Thin Film through Post-Modification
Source: Nanomaterials (Basel). 2017 Aug 29;7(9):242. doi: 10.3390/nano7090242 (PMC5618353; doi:10.3390/nano7090242)
Supplement: Supplementary file 1 [file nanomaterials-07-00242-s001.pdf]

**Supplementary Materials**

# The tuning of optical properties of nanoscale MOFs-based thin film through post-modification

**Wenchang Yin,[1] Cheng-an Tao,[1,*] Xiaorong Zou,[1] Fang Wang,[1] Tianlian Qu,[2] Jianfang Wang[1,*]**

[1] College of Science, National University of Defense Technology, Changsha 410073, China; yinwenchang8@163.com(W. Y.), tca02@mails.thu.edu.cn (C. T.), Xr5229@163.com(X. Z.), wangaaf02@163.com(F. W.), wangjianfang@nudt.edu.cn(J. W.)

[2] College of Optoelectronic Science and Engneering, National University of Defense Technology, Changsha 410073, China; qutianliang@nudt.edu.cn(T. Q.)

* Correspondence: tca02@mails.thu.edu.cn; Tel.: +86-731-8700-2334 (C. T.); wangjianfang@nudt.edu.cn; Tel.: +86-731-8457-4241 (J. W.)

## 1. Synthesis and Characterization of nanoscale $NH_2$-MIL-53(Al)

### *1.1 Materials*

$Al(NO_3)_3 \cdot 9H_2O$ (97%), 2-aminoterephthalic acid ($H_2N$-BDC, 99%), propionaldehyde (C3), pentanal (C5) and heptanal (C7) were purchased from J&K chemical. All the other chemicals are products of local chemical. All reagents were used as received without further purification. Deionized water was used throughout the work. Silicon wafers (2 cm × 2 cm) were used as substrates and were pre-cleaned with soap and water and subsequently treated with Piranha solution ($H_2SO_4/H_2O_2$, volumetric ratio 7:3). After thoroughly rinsing with deionized water, the wafers were dried under hot air flow and stored in ethanol.

### *1.2 Synthesis of $NH_2$-MIL-53(Al) Nano Rods*

2-aminoterephthalic acid (0.9417g, 5.20 mmol) was added to a 50 mL centrifuge tube. Then sodium acetate trihydrate (1.4370 g, 10.56mmol) was added. $Al(NO_3)_3.9H_2O$ (1.320 g, 3.52 mmol) was added to a separate 50 mL centrifuge tube. The volume in each tube was increased to a total of 25 mL by addition of $H_2O$. Both tubes were sonicated for 30 minutes to ensure even dispersion. Then, contents from both tubes were mixed in a 100 mL Teflon cup-lined autoclave, sealed and left to react in an oven at 120$^{o}$C for 72 hrs. The resulting mixture was collected by centrifugation at 12000 rpm for 10 minutes. The supernatant was decanted leaving yellowish solid. The solid was washed with acetone and collected by centrifugation twice. The resulting yellowish solid was purified by refluxing in methanol at 90$^{o}$C for 12 hrs. The resulting

material was collected by centrifugation, washed with ethanol twice, then centrifuged again and dispersed the $NH_2$-MIL-53(Al) NRs in ethanol for the fabrication of optical thin film and other characterization.

### *1.3 Characterization*

**TEM:** TEM micrographs were obtained using a JEOL JEM 1230 electron microscope with an accelerating voltage of 200 kV. Samples for the TEM observation were prepared by dropping 6 μL of $NH_2$-MIL-53(Al) suspension.

**FT-IR:** Fourier transform infrared (FT-IR) spectra were obtained using a Spectra Two spectrophotometer(PerkinElmer) within the spectral range of 4000 $cm^{-1}$ to 400 $cm^{-1}$ with attenuated total reflection (ATR) accessory. Samples were obtained by purifying the products with methanol at 90℃ for 12 hrs, drying the alcoholic suspensions at 90℃ overnight, and finally with a activating treatment at 330℃ in air for 6 hrs.

**XRD:** Powder X-ray data were collected using a Ttr III type X-ray diffractometer (Rigaku, Japan) in θ–θ geometry from 5° to 50° (2θ) with a graphite-monochromated CuKα radiation source. The samples were held in a non-reflective holder stage and scanned by 6 °/min scan speed in continuous mode. Before the measurement, the yellow power samples purified by methanol were activated at 330 °C in air for 6 hrs.

**TGA:** Thermogravimetric analyses (TGA) were performed on a STA6000 thermogravimetric analyzer (Perkin Elmer). In order to remove the unreacted 2-aminoterephthalic acid as much as possible, the samples were refluxed in DMF for

12hrs after purified by methanol, then washed with ethanol to remove DMF. After dried in air at 90℃ overnight, the samples were measured under nitrogen using a ramp rate of 5℃ per min from 30℃ to 700℃.

**$N_2$ Ads-des:** $N_2$ adsorption-desorption isotherms were carried out using BEL Mini sorption. The BET area of NRs calculated between $P/P_0$ 0.01 and 0.25. Sample was obtained with the same treatment as that prepared for FT-IR test.

## 2. Ellipsometry measurements of optical films

Ellipsometry measurements were carried out using a XLS-100 ellipsometer (Woollam) at angles of 65°,70° and 75° and within a spectral range of 400-1000 nm at room temperature. In order to get the refractive index data, we used the Wvase 32 software to fit the $\psi$ and $\Delta$ data got from the ellipsometry measurements. During the fitting process, we made Si as the basic layer and added a user's layer which stands for MOFs optical film. Then we chose the Cauchy model which is suitable for uniform thin film to obtain the refractive index data and the thickness of the films.

The generated and experimental Psi &Delta plots for MOFs optical films were shown in Figure S2-S5.

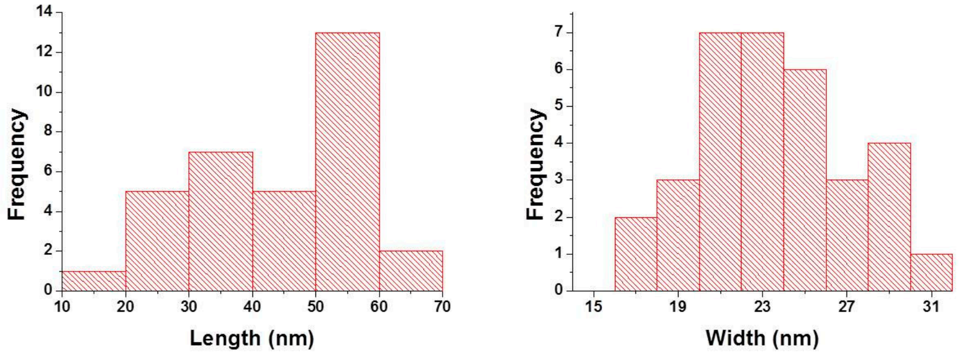


**Figure S1.** Statistic histograms of length and width of MOFs nanorods in the TEM image in figure 1A

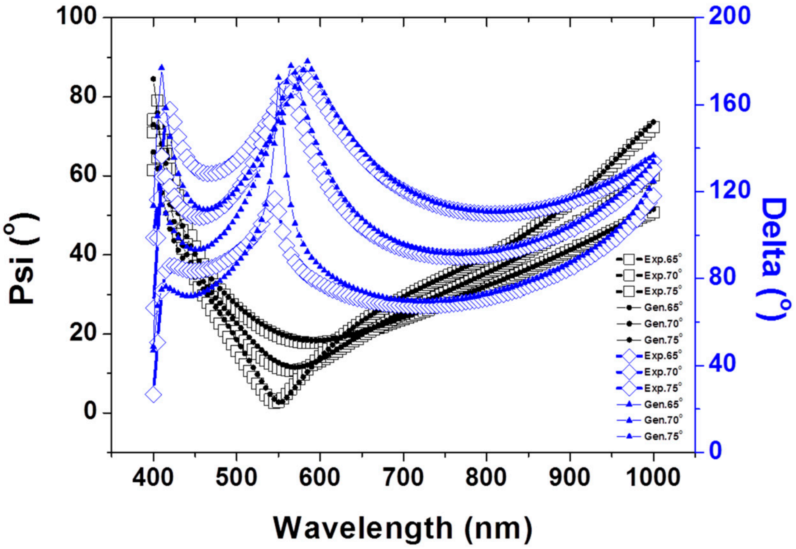


**Figure S2.** Generated and Experimental Psi &Delta plots for MOFs optical film with no modification

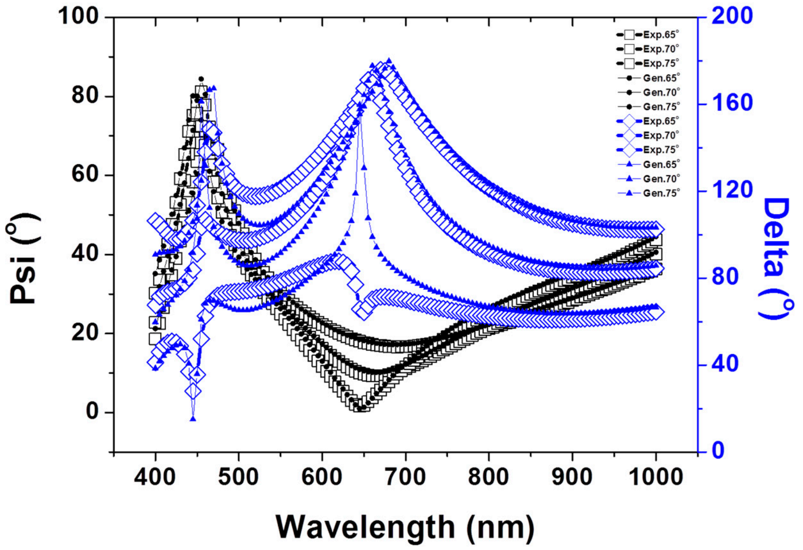


**Figure S3.** Generated and Experimental Psi &Delta plots for MOFs optical film modified with C3

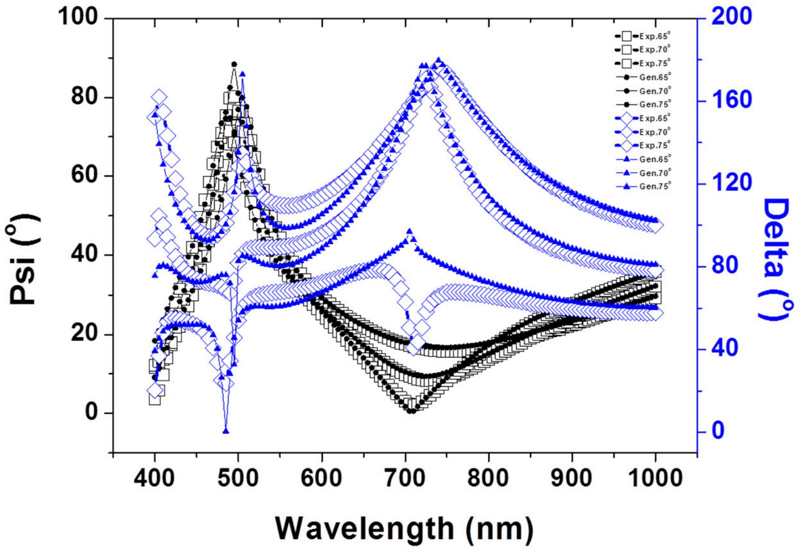


**Figure S4.** Generated and Experimental Psi &Delta plots for MOFs optical film modified with C5

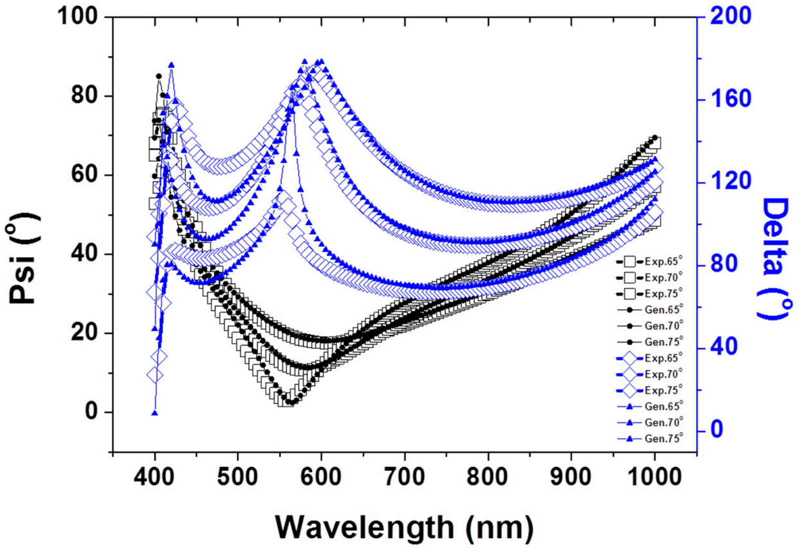


**Figure S5.** Generated and Experimental Psi &Delta plots for MOFs optical film modified with C7

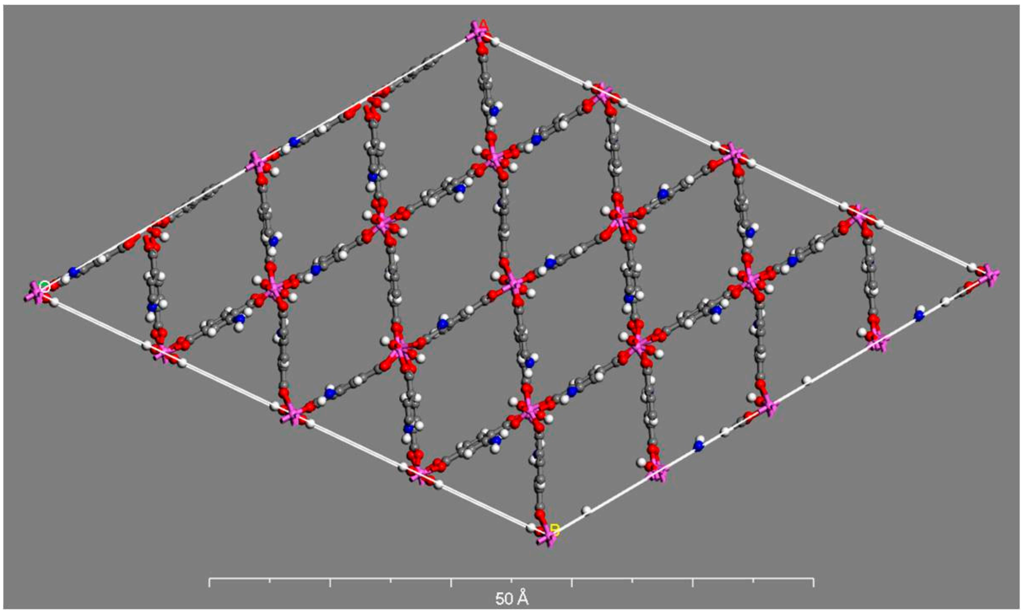


**Figure S6.** Crystalline structure of $NH_2$-MIL-53(Al) generated from the CIF of $NH_2$-MIL-53(Al) [**1**] with Diamond software.

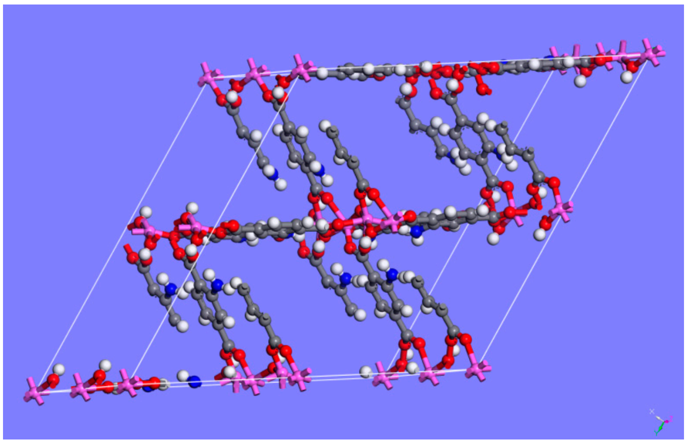

**Figure S7.** Crystalline structure of $NH_2$-MIL-53(Al) generated from the CIF of $NH_2$-MIL-53(Al) [1] with Diamond software (View in another direction)

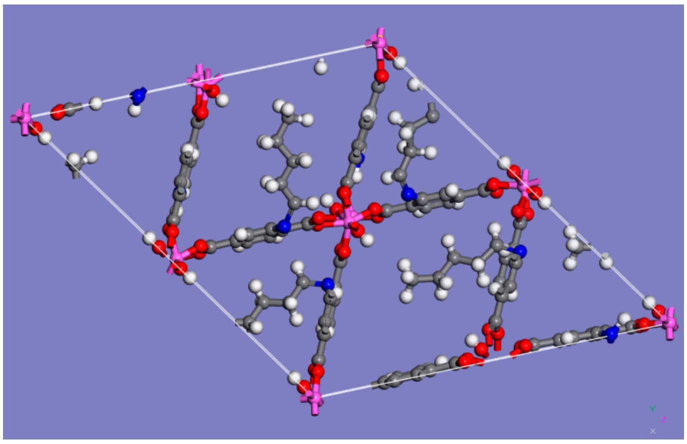

**Figure S8.** Illustration of the alkyl chains in the channel of MOFs, C5 is taken as an example.

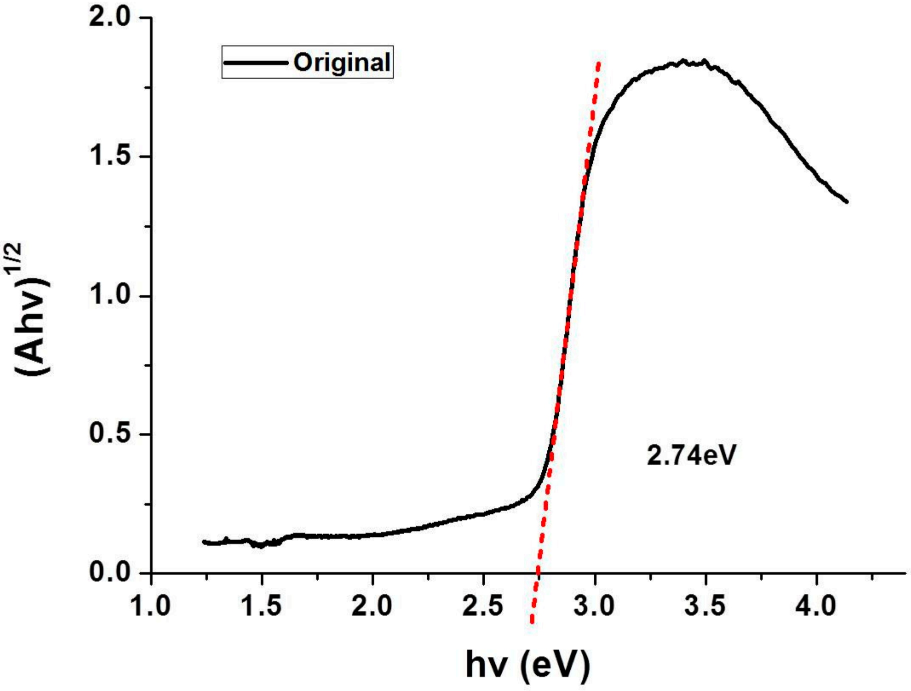


**Figure S9.** The optical band gaps of $NH_2$-MIL-53(Al) NRs

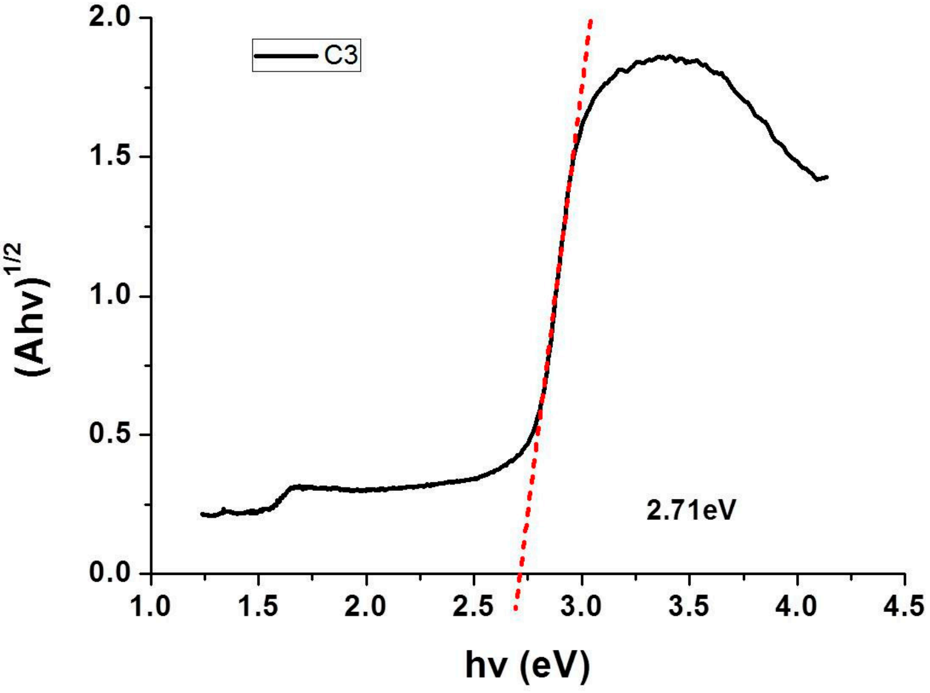


**Figure S10.** The optical band gaps of $NH_2$-MIL-53(Al) NRs modified with C3

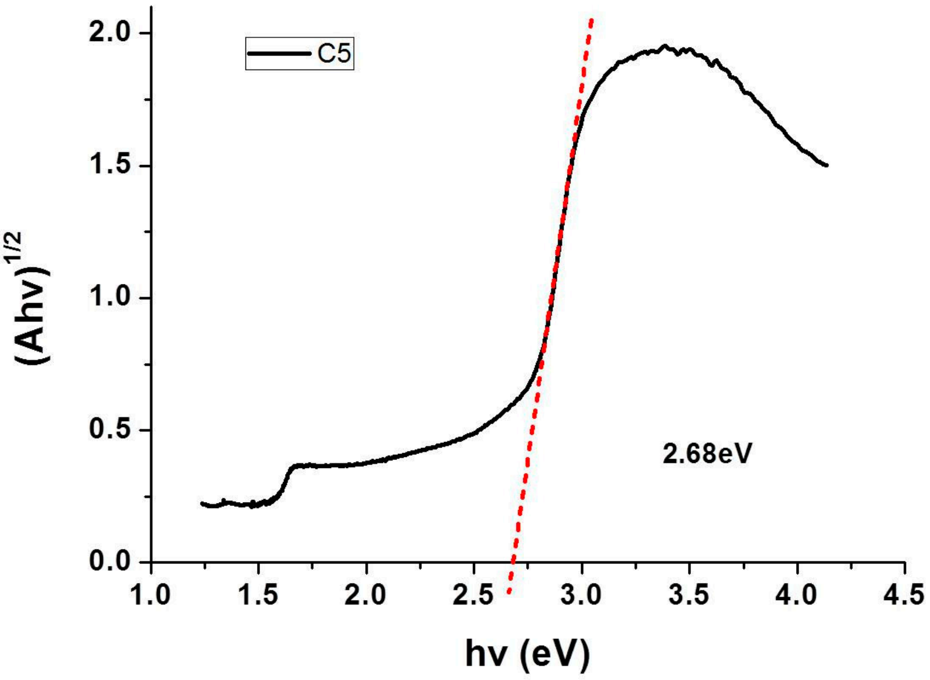


**Figure S11.** The optical band gaps of $NH_2$-MIL-53(Al) NRs modified with C5

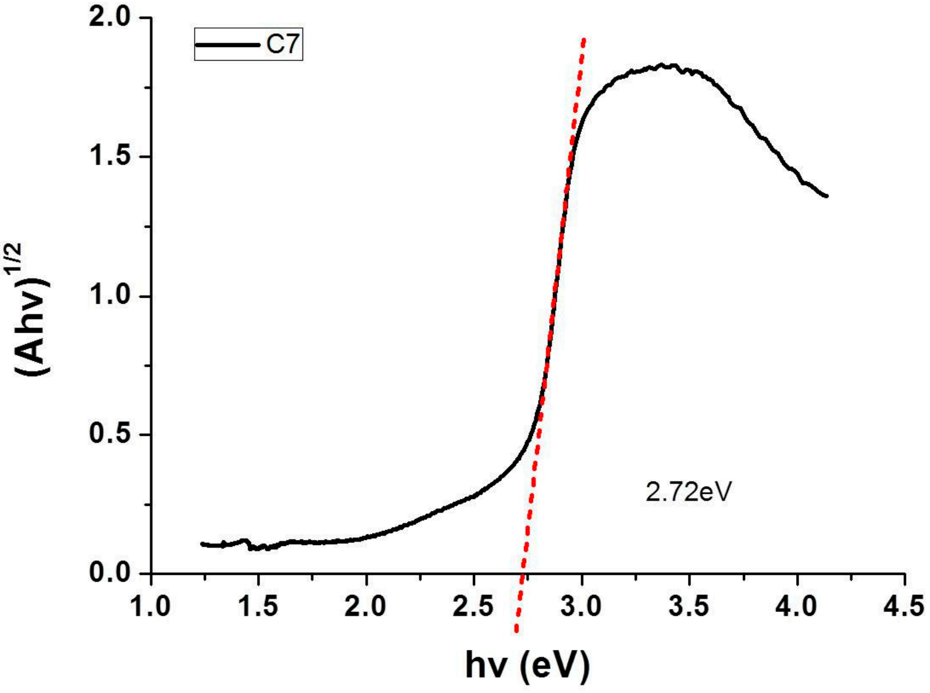


**Figure S12.** The optical band gaps of $NH_2$-MIL-53(Al) NRs modified with C7

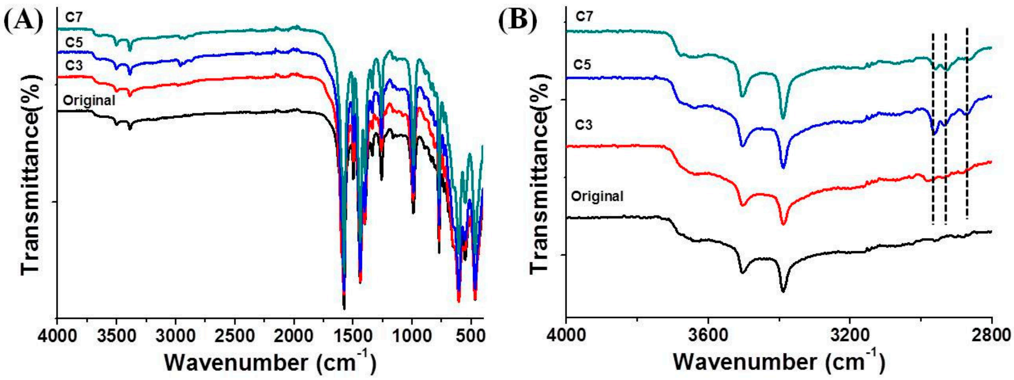


**Figure S13.** (A) FT-IR of MOFs without modification, modified with propionaldehyde (C3), pentanal (C5) and heptanal (C7). (B) The enlarged FT-IR from 4000 cm$^{-1}$ to 2800 cm$^{-1}$ of (A).

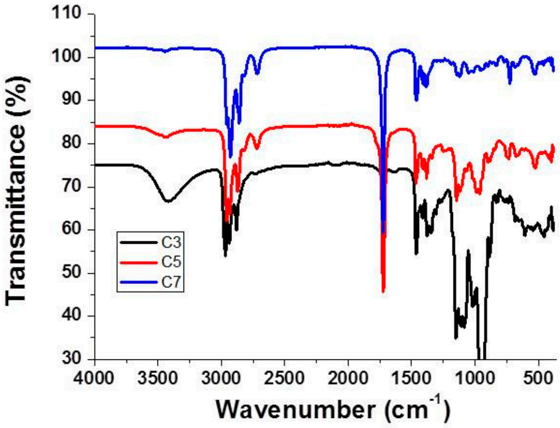


**Figure S14.** FT-IR of propionaldehyde (C3), pentanal (C5) and heptanal (C7).

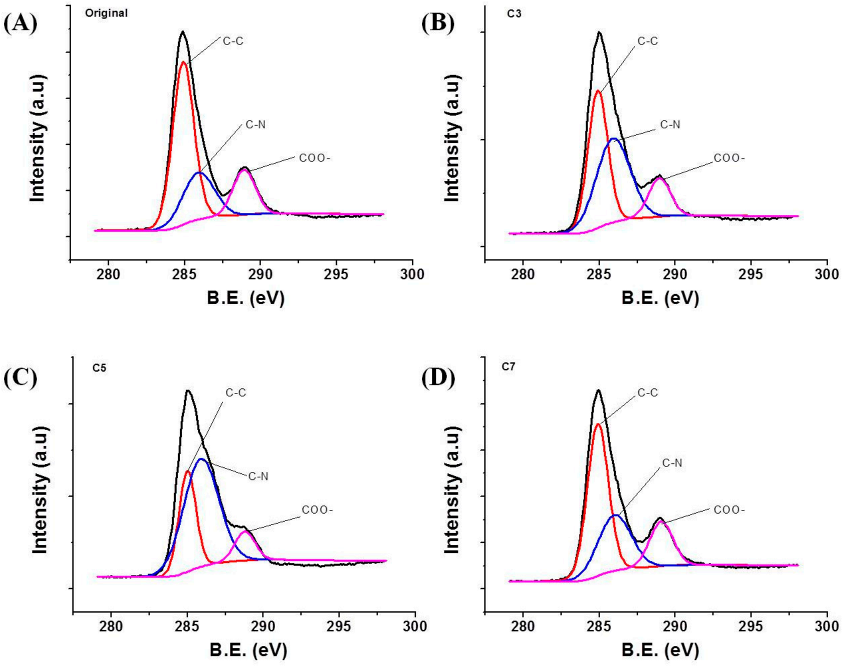


**Figure S15.** The C1s XPS spectra fitted with three peaks of C-C, C-N and $COO^-$ for MOFs optical films without post-modification(A) , modified with C3 (B) , C5 (C) and C7 (D).

**Table S1** The atomic percent of C in different chemical groups evaluated from fitted peaks of C1s XPS spectra

| | C-C (%) | C-N (%) | COO (%) | C-N/COO |
|---|---|---|---|---|
| Original | 58.8 | 24.5 | 16.7 | 1.5 |
| C3 | 43.0 | 44.0 | 13.0 | 3.4 |
| C5 | 29.3 | 60.7 | 9.9 | 6.1 |
| C7 | 53.1 | 29.9 | 16.9 | 1.8 |

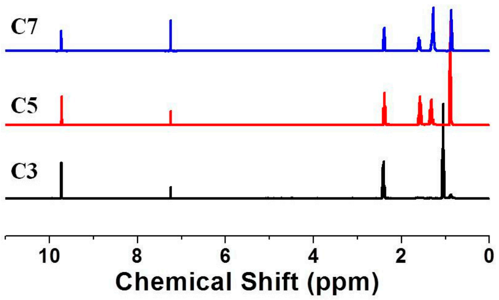


**Figure S16.** $^1$H-NMR spectra of C3, C5 and C7 molecules in $CDCl_3$.

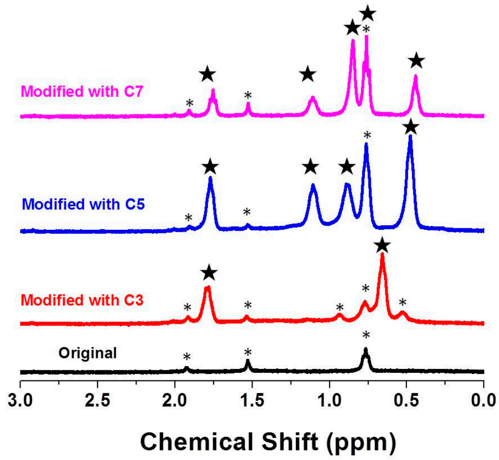


**Figure S17.** $^1$H-NMR spectra of MOFs digested in NaOD-$D_2O$ solution. From bottom to top: Original MOFs, modified with C3, C5 and C7. Solid pentastars (★) and stars (*) represent signals of alkyl and impurities, respectively.

**Table S2.** Degree of modification of MOFs with different aldehydes

| Modifier | Degree(%) From XPS | Degree(%) From $^1$H NMR |
|---|---|---|
| C3 | 25.3% | 47.2% |
| C5 | 42% | 50.9% |
| C7 | 14.6% | 28.0% |

[1] Jia Min Chin, Eric Yu Chen, Ajay Govinda Menon, Han Yang Tan, Andy Tzi Sum Hor, Martin Karl Schreyer and Jianwei Xu. Tuning the aspect ratio of $NH_2$-MIL-53(Al) microneedles and nanorods via coordination modulation. CrystEngComm, 2013, 15, 654.
